# A rare case of recurrent primary dumbbell-shaped spinal hydatidosis^☆^

**DOI:** 10.1016/j.radcr.2022.06.004

**Published:** 2022-07-01

**Authors:** Xin Xu, Jiancang Cao, Jiabing Wang, Tingting Wu, Ping Xu

**Affiliations:** a Lanzhou University Second Hospital, Lanzhou, Gansu 730000, China; b The first Clinical Medical College of Gansu University of Chinese Medicine, Gansu 730000, China; c Gansu Provincial Hospital, Gansu 730000, China

**Keywords:** Spine, Echinococcosis, Magnetic resonance imaging

## Abstract

Spinal hydatidosis, which affects the thoracic vertebrae, is not only an extremely rare occurrence, but is also characterized by a high recurrence rate. Here, we reported a case of 67-years-old man who presented with recurrent spinal hydatid disease. The condition was originally misdiagnosed as Schwannoma via medical imaging, but eventually confirmed by postoperative pathology. He was subjected to surgery, combined with adjuvant drug therapy. Unfortunately, he experienced recurrent spinal hydatid disease and had to undergo hydatid cyst excision in over 5 years.

## Introduction

Spinal hydatidosis is an extremely rare occurrence in both children and adults. Previous studies have shown that incidence of vertebral column involvement among patients with hydatidosis is 1%, while bone infestation is approximately 45% [1]. Radiologic diagnosis of vertebral hydatidosis, which commonly involves the thoracic vertebra [2], is often difficult. Although surgery is an aggressive treatment therapy for the disease, the recurrent rate remains high. Therefore, it is an imperative management to combine the routine medical imaging with clinical history in the preoperative evaluation and during follow up. In this report, we described a rare primary paravertebral spinal hydatidosis with intraspinal extension. The patient underwent 2 operations. We followed up the case for 5 years, thus we discussed our experiences in the diagnosis of spinal hydatidosis.

## Case report

**Fig. 1:**
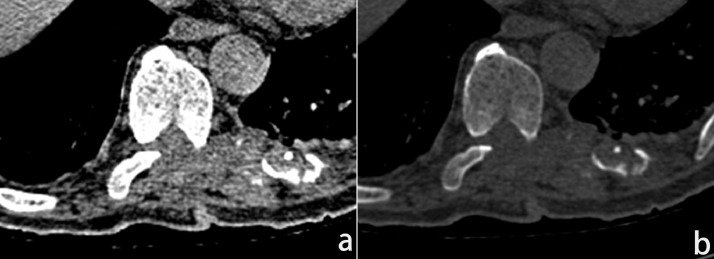
(a) CT soft tissue window. (b) CT bone window.

**Fig. 2:**
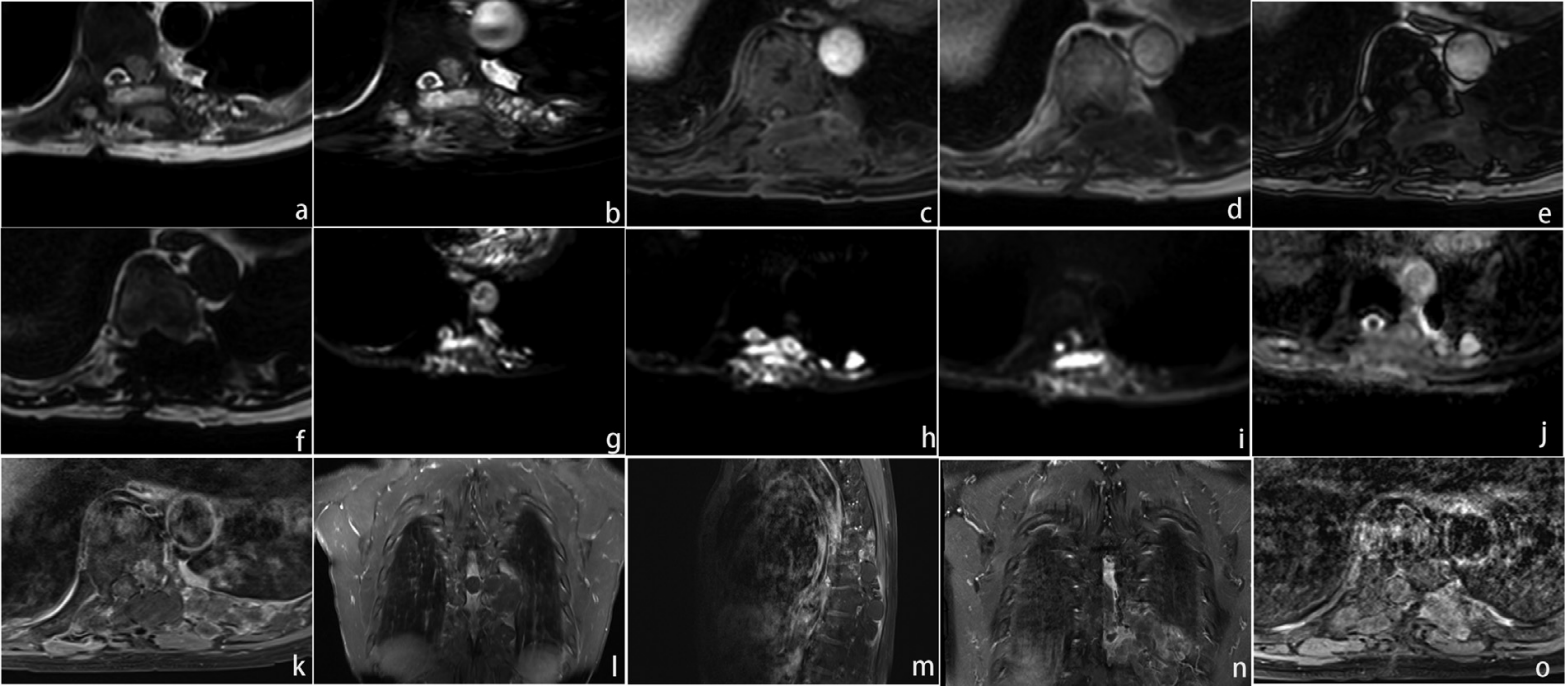
(a) MRI T2WI (axis image) showing hyperintense signal of a multilocular cystic cavity at the left side of the vertebral body. (b) MRI T2FS showing hyperintense of the cavity content. (c) TIWI Dixon sequence: WO image showing isointense signal of the lesion. (d) IP image showing hypointense. (e) OP image. (f) FO image showing hypointense. (g) MRI DWI (b = 0). (h) DWI (b = 600). (i) DWI (b = 1000). (g–i) showing hyperintense of the cavity. (j) MRI ADC map showing isointense. (k) Enhancement MRI (axis scan) showing the cystic cavity content without enhancement and the wall with slight enhancement. (l) Enhancement MRI (coronal scan) showing the cystic lesions in the spinal canal no enhancement. (m) Enhancement MRI (sagittal scan) showing the lesion ranges from T8 to T10 level. (n) Enhancement MRI (coronal scan, 2021) showing the lesion is larger, and the boundary with surrounding tissues unclear. (o) Enhancement MRI (axis scan, 2021) showing the cavity with more obvious enhancement.

A 67-years-old man presented at Lanzhou University Second Hospital with complaints of lumbago and numbness in both lower extremities for 2 months. Before the first surgery (conducted in 2016), results from computed tomography (CT) scan showed soft tissue extension of the lesion, the relevant intervertebral was enlarged, while the relevant vertebral body appendix and adjacent rib were destroyed (Figs. 1a and b). T2-weighted-images (T2WI) and T2-fat-suppression-image (T2FS) revealed that the cyst content in the lesion was not only hyperintense, but was also surrounded by low-intensity rim on (Figs. 2a and b). Moreover, T1-weighted-images (T1WI) Dixon sequence revealed the tumour without suppression of the subcutaneous fat. These sequences are as follows: Water-only (WO) (Fig. 2c), In-phase (IP) (Fig. 2d), out-of-phase (OP) (Fig. 2e), fat only (FO) (Fig. 2f). Diffusion-weighted-imaging (DWI) and isointense on apparent-diffusion-conversion (ADC) revealed that the lesion was hyperintense (Figs. 2g and j). Furthermore, enhanced MRI revealed that the multiloculated cystic lesions originated from the T8-T10 left paraspinal space, widened on the left T9-T10 neural foramen extending to T9 spinal canal, then produced a dumbbell-shaped appearance. The cystic lesion exhibited a clear boundary with surrounding tissues, and the cystic cavity content was not enhanced, but the cystic wall was slightly enhanced (Figs. 2k–m). Ultrasound scan revealed normal results. Unfortunately, according to the characteristics of CT and MRI imaging, the disease was misdiagnosed as Schwannoma. Consequently, the patient underwent an operation.

**Fig. 3:**
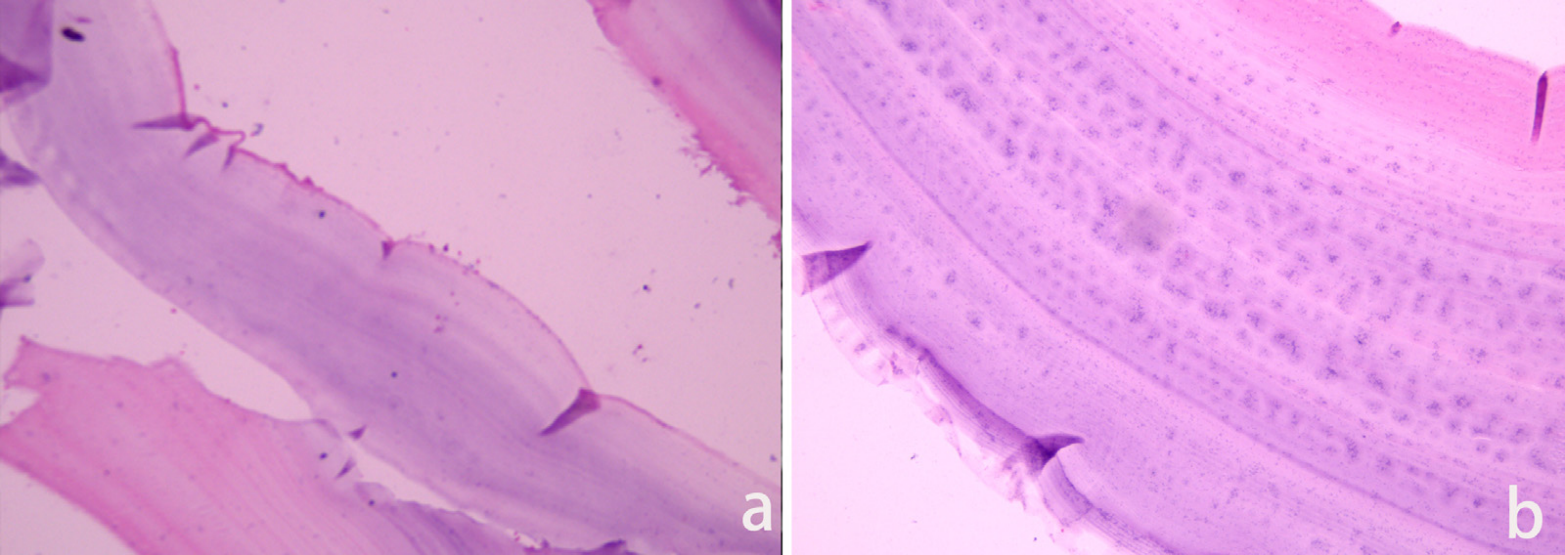
(a and b) Histopathological examination (Hematoxylin-Eosin staining): A thin membranous structure consisting of the outer and inner sacs can be seen (a) original magnification × 100. (b) Original magnification × 400).

Recently, the spinal hydatid cyst recurred, and upon physical examination, it was evident that all forms of sensations in the lower limbs had decreased bilaterally. Results from local examination revealed a prominent midline scar in the back region. Notably, liver and renal function tests, as we as coagulation tests, were normal. Ultrasonography revealed many cysts in the liver, while MRI revealed multiple variably sized cysts swelling in left paraspinal area of T8-T10 levels and the cyst walls were slightly enhanced. There were neither clear boundaries between the cysts and surrounding tissues, nor evidence of spinal cord compression (Figs. 2n and o). The patient underwent surgery again, and surgical pathology revealed hydatidosis (Figs. 3a and b). Postoperation, he was put on combination treatment of albendazole. He recovered, 1 week after the operation, uneventfully, and without any neurological deficits.

## Discussion

Hydatidosis has a global distribution, and is found in every continent, especially in pastoral area. The disease principally occurs in dogs, wolves, foxes, sheep, mice, horses [3]. Humans are only accidental intermediate hosts, and cannot transmit the disease. In endemic regions, the incidence of this disease in humans can reach greater than 0.05% per year [4]. Notably, hydatidosis infection in humans mainly occurs in the liver and lungs, and the disease has a very long period of asymptomatic incubation until hydatid cysts grow to an extent that results clinical symptoms [5]. The classical presentation of this disease is spinal cord compression. Moreover, several retrospective studies have reported the rate of medullary compression in case of spine hydatidosis ranges between 47% and 73% [6].

Diagnosis of hydatidosis not only depends on serological examinations, but also on radiology and biology. Certainly, histopathological diagnosis is the gold standard. In the present case, our patient had undergone 2 previous serological tests that returned negative results. Although CT can be used to assess bony involvement, it is not suitable for identification of soft tissue components. Notably, MRI is the preferred imaging technique for diagnosis of spinal hydatidosis. Some unique appearances have been shown in the spinal hydatid. In the present case, MRI revealed a multiloculated cyst hypointense on T1WI and hyperintense on T2WI. The cyst walls were thin and regular, with no evidence of calcification and a slight enhancement. The diffusion coefficient restricted on DWI in conjunction with ADC map. The recurrent lesion was larger and more significant than previous results from enhanced MRI. We attributed the difference to bone destruction or disease progression, in addition to the formation of scar tissue after the operation. The misdiagnosis, as Schwannoma, may have been due to the similarity of spinal hydatidosis on MRI and without pastoral area residence. Image analysis of this patient indicated that the posterior edge of vertebral had been defected but without sclerosis, which is important distinguishing hydatidosis from Schwannoma. Although surgery is an aggressive treatment therapy, it cannot completely cure spinal hydatidosis because of the absence of anatomical features and the existence of neural structures [7]. The mortality rate is almost 100% for the multilocular form [8].

## Conclusions

Patients with spinal hydatidosis usually present with symptoms of mechanical compression of the spinal cord. Technological advancement in medical imaging has enabled early detection and early removal of tumors, thereby improving patient prognosis. Once medical imaging reveals numerous cysts inside the spinal canal and the patient has an endemic area life history, the disease should be considered spinal hydatidosis. Although CT and MRI techniques play a crucial role in the positioning, qualitative, and quantitative diagnosis of this disease, follow up is also needed to detect and take care of any potential recurrence.

## Ethical approval

We further confirm that the case collection covered in this manuscript that has involved our patient that has been approved by the ethics committee of Lanzhou University Second Hospital. All procedures were performed in accordance with the ethical standards of the institutional research committee and with the Helsinki declaration.

## Patient consent statement

Written informed consent was obtained from the patient.

## Appendix Supplementary materials

Image, application 1
