# Supplementary material for: A rare case of recurrent primary dumbbell-shaped spinal hydatidosis
Source: Radiol Case Rep. 2022 Jul 1;17(9):3224–7. doi: 10.1016/j.radcr.2022.06.004 (PMC9256548; doi:10.1016/j.radcr.2022.06.004)
Supplement: Supplementary file 1 [file mmc1.doc]

**Figure 1: a**. CT soft tissue window. **b**. CT bone window. **c**. MRI T2WI (axis image) showing hyperintense signal of a multilocular cystic cavity at the left side of the vertebral body. **d**. MRI T2FS showing hyperintense of the cavity content. **e**. TIWI Dixon sequence: WO image showing isointense signal of the lesion. **f**. IP image showing hypointense. **g**. OP image. **h**. FO image showing hypointense. **i**. MRI DWI(b=0). **j**. DWI(b=600). **k**. DWI(b=1000). i,j,k showing hyperintense of the cavity. **l**. MRI ADC map showing isointense. **m**. Enhancement MRI (axis scan) showingthe cystic cavity content without enhancement and the wall with slight enhancement. **n**. Enhancement MRI(coronal scan) showing the cystic lesions in the spinal canal no enhancement. **o**. Enhancement MRI(sagittal scan) showing the lesion ranges from T8 to T10 level. **p**. Enhancement MRI(coronal scan, 2021) showing the lesion is larger, and the boundary with surrounding tissues unclear. **q**. Enhancement MRI(axis scan, 2021) showing the cavity with more obvious enhancement. **r, s** Histopathological examination(Hematoxylin-Eosin staining): A thin membranous structure consisting of the outer and inner sacs can be seen.
